# Differential proteomic analysis of synovial fluid from rheumatoid arthritis and osteoarthritis patients

**DOI:** 10.1186/1559-0275-11-1

**Published:** 2014-01-06

**Authors:** Lavanya Balakrishnan, Mitali Bhattacharjee, Sartaj Ahmad, Raja Sekhar Nirujogi, Santosh Renuse, Yashwanth Subbannayya, Arivusudar Marimuthu, Srinivas M Srikanth, Rajesh Raju, Mukesh Dhillon, Navjyot Kaur, Ramesh Jois, Vivek Vasudev, YL Ramachandra, Nandini A Sahasrabuddhe, TS Keshava Prasad, Sujatha Mohan, Harsha Gowda, Subramanian Shankar, Akhilesh Pandey

**Affiliations:** 1 Institute of Bioinformatics, International Technology Park, Bangalore 560066, India; 2 Department of Biotechnology, Kuvempu University, Shankaraghatta 577451, India; 3 Amrita School of Biotechnology, Amrita Vishwa Vidyapeetham, Kollam 690525, India; 4 Manipal University, Madhava Nagar, Manipal 576104, India; 5 Centre for Excellence in Bioinformatics, School of Life Sciences, Pondicherry University, Puducherry 605014, India; 6 Rajiv Gandhi University of Health Sciences, Bangalore 560041, India; 7 Department of Internal Medicine, Armed Forces Medical College, Pune 411040, India; 8 Department of Rheumatology, Fortis Hospitals, Bangalore 560076, India; 9 Department of Rheumatology, Command Airforce Hospital, Bangalore 560008, India; 10 Laboratory for Integrated Bioinformatics, RIKEN Center for Integrative Medical Sciences (IMS-RCAI), Yokohama Institute, Kanagawa 230-0045, Japan; 11 McKusick-Nathans Institute of Genetic Medicine, Johns Hopkins University School of Medicine, Baltimore, MD 21205, USA; 12 Department of Oncology, Johns Hopkins University School of Medicine, Baltimore, MD 21205, USA; 13 Department of Pathology, Johns Hopkins University School of Medicine, Baltimore, MD 21205, USA; 14 Department of Biological Chemistry, Johns Hopkins University School of Medicine, Baltimore, MD 21205, USA

**Keywords:** Arthritis, Joint inflammation, Cartilage degradation, Extracellular matrix

## Abstract

**Background:**

Rheumatoid arthritis and osteoarthritis are two common musculoskeletal disorders that affect the joints. Despite high prevalence rates, etiological factors involved in these disorders remain largely unknown. Dissecting the molecular aspects of these disorders will significantly contribute to improving their diagnosis and clinical management. In order to identify proteins that are differentially expressed between these two conditions, a quantitative proteomic profiling of synovial fluid obtained from rheumatoid arthritis and osteoarthritis patients was carried out by using iTRAQ labeling followed by high resolution mass spectrometry analysis.

**Results:**

We have identified 575 proteins out of which 135 proteins were found to be differentially expressed by ≥3-fold in the synovial fluid of rheumatoid arthritis and osteoarthritis patients. Proteins not previously reported to be associated with rheumatoid arthritis including, coronin-1A (CORO1A), fibrinogen like-2 (FGL2), and macrophage capping protein (CAPG) were found to be upregulated in rheumatoid arthritis. Proteins such as CD5 molecule-like protein (CD5L), soluble scavenger receptor cysteine-rich domain-containing protein (SSC5D), and TTK protein kinase (TTK) were found to be upregulated in the synovial fluid of osteoarthritis patients. We confirmed the upregulation of CAPG in rheumatoid arthritis synovial fluid by multiple reaction monitoring assay as well as by Western blot. Pathway analysis of differentially expressed proteins revealed a significant enrichment of genes involved in glycolytic pathway in rheumatoid arthritis.

**Conclusions:**

We report here the largest identification of proteins from the synovial fluid of rheumatoid arthritis and osteoarthritis patients using a quantitative proteomics approach. The novel proteins identified from our study needs to be explored further for their role in the disease pathogenesis of rheumatoid arthritis and osteoarthritis.

Sartaj Ahmad and Raja Sekhar Nirujogi contributed equally to this article.

## Background

Rheumatoid arthritis (RA) is a common systemic autoimmune disorder. Around 0.5-1% of the adult population is affected with RA in the developed countries with 5–50 per 100,000 new cases reported annually [1]. RA is characterised by persistent inflammation of the synovial membrane and pannus formation that results in joint damage and loss of function [1,2]. Genetic, environmental and stochastic factors act together to contribute to the pathogenesis of RA [3]. Osteoarthritis (OA) is a progressive disorder characterized by the degradation of the cartilage, osteophyte formation, mild to moderate synovial inflammation, narrowing of the joint space and subchondral sclerosis [4,5]. It is one of the most prevalent musculoskeletal diseases that lead to disability in ~40% of the adults over 70 years [5].

Despite significant advances towards the understanding of the pathophysiology of RA and OA, early diagnosis and therapeutic intervention remain a challenge [6]. RA is usually diagnosed based on clinical symptoms and the presence of antibodies against rheumatoid factor (RF) and cyclic citrullinated peptides (CCPs) in serum [7]. Although RF has been traditionally used as a biomarker for RA, it lacks specificity as it is also detected in the sera of several other autoimmune disorders and infectious diseases as well as in the healthy elderly population (10-30%) [7,8]. Antibodies to CCPs have been shown to be involved in the development of autoimmune arthritis [9]. Their specificity for RA has been reported to be higher than RF. However the diagnostic sensitivity of antibodies to CCPs has been estimated to be slightly lower than that of RF [10]. In addition to anti-CCPs, anti-filaggrin (AFA) and anti-Sa antibodies have also been demonstrated to have high specificity for RA but with a sensitivity of less than 50% [11]. Assessment of the pathological changes in the OA joint is mainly carried out using radiography, which is the gold standard for diagnostic purposes [12]. However, it has poor sensitivity as the radiographic evidence is obtained only when the articular cartilage has degraded significantly. Thus, this does not provide an account of the extent of disease progression [12]. Biomarkers that are useful for early diagnosis or for predicting the outcome/progression of OA are not currently available [13]. Thus, there is a need for continued discovery efforts to identify novel biomarkers with the desired sensitivity and specificity for RA and OA.

Mass spectrometry-based approaches have provided an impetus for biomarker discovery for a wide range of diseases. Studying the synovial fluid proteome is beneficial in arthritis as it is in proximity to the site of disease activity as well as it provides a snapshot of the most relevant compartment throughout disease progression. Additionally, alterations in the joint cavity due to injury or disease may be directly reflected in the composition of synovial fluid and could be correlated to disease severity and progression [14]. In a previous proteomic study, surface enhanced laser desorption ionization (SELDI) mass spectrometry was used to identify proteins that were present in RA synovial fluid but not in OA [15]. Subsequently, in another proteomic study, 2-D gel electrophoresis followed by MALDI-TOF analysis was carried out to identify synovial fluid and plasma proteins that could differentiate between RA and reactive arthritis (ReA) or OA. S100A9, S100A12 and serum amyloid A (SAA) were found to be present only in RA but not in ReA and OA in that study [16]. A label free quantitative analysis of RA and OA synovial fluid proteome by Mateos *et al*., resulted in the identification of 135 proteins using MALDI-TOF/TOF mass spectrometer [17]. Proteins involved in complement activation, inflammation and immune response were found to be relatively more abundant in RA and those that participated in the extracellular matrix formation and remodeling were more abundant in OA synovial fluid [17]. An immunoproteomics study has also been reported to identify autoantigens in RA. In this study, the expression of vimentin, gelsolin, alpha-2-HS-glycoprotein, glial fibrillary acidic protein and alpha-1-B glycoprotein was found to be reported to be higher in RA synovial fluid than that of OA [18]. Most of the studies carried out thus far have adopted a label free quantitation approach and employed low resolution mass spectrometers to identify the proteins differentially expressed between RA and OA.

Isobaric Tags for Relative and Absolute Quantification (iTRAQ) is a chemical labeling method which uses isobaric tags with reporter ion group that react with the primary amine groups present in the peptides [19]. iTRAQ-based quantitative proteomics approach coupled to mass spectrometry has been widely used to identify biomarkers for several diseases including cancer [20]–[24], meningitis [25] and rabies [26]. Here, we describe an iTRAQ-based strategy to relatively quantitate the synovial fluid proteins from RA and OA. We employed an iTRAQ-based labeling strategy coupled with LTQ-Orbitrap Velos mass spectrometer to identify proteins differentially expressed between RA and OA. The differentially expressed proteins obtained from the study might increase our knowledge pertaining to the mechanism of pathogenesis of RA and OA.

## Results and discussion

**Figure 1:**
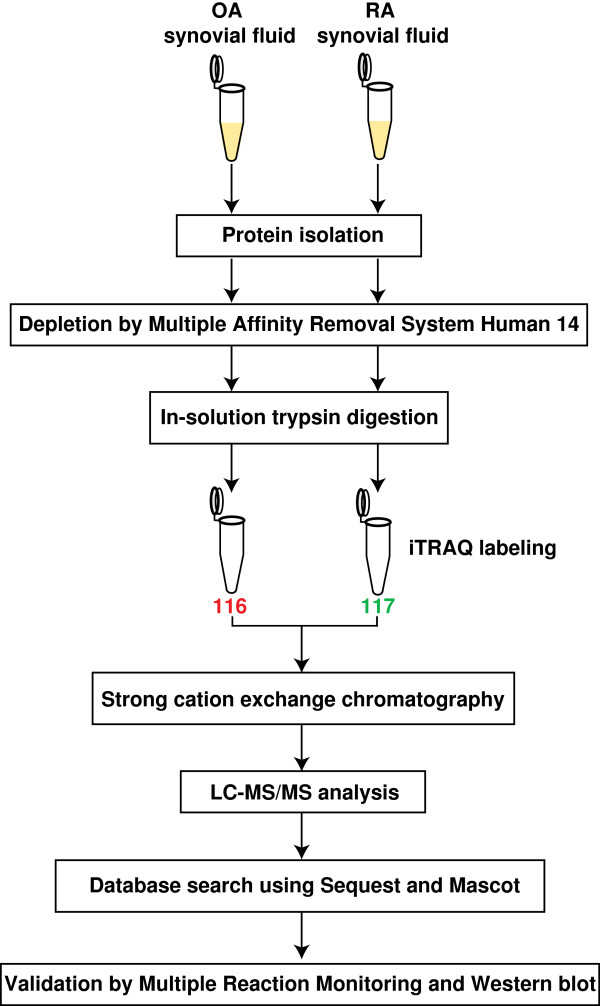
**A schematic workflow illustrating the steps involved in the differential analysis of RA and OA synovial fluid proteome.** Proteins from RA and OA synovial fluid were extracted and depleted to remove the 14 most abundant proteins using multiple affinity removal system, Human-14. The depleted protein from RA and OA were then digested with trypsin and labeled with iTRAQ reagents, 117 and 116 respectively. The labeled samples were pooled and subjected to fractionation using strong cation exchange chromatography. The fractions were then analyzed on a LTQ-Orbitrap Velos mass spectrometer. The MS/MS data obtained was searched against Human RefSeq 50 database using Sequest and Mascot search algorithms. Validation of the iTRAQ quantitation data was carried out using multiple reaction monitoring and Western blot.

We employed a quantitative proteomics approach using iTRAQ labeling to identify the differentially expressed proteins in RA compared to OA. A schematic workflow illustrating the steps employed in this study is shown in Figure 1.

### Quantitative proteomic analysis of the synovial fluid proteome of RA and OA

A total of 30,226 MS/MS spectra acquired from the LC-MS/MS analysis of 20 SCX fractions resulted in the identification of 3,488 unique peptides corresponding to 575 proteins. Out of these 575 proteins, 135 were differentially expressed between RA and OA by ≥3-fold. A complete list of all the identified peptides along with their corresponding proteins is provided in Additional file 1: Table S1.

### Bioinformatics analysis

**Figure 2:**
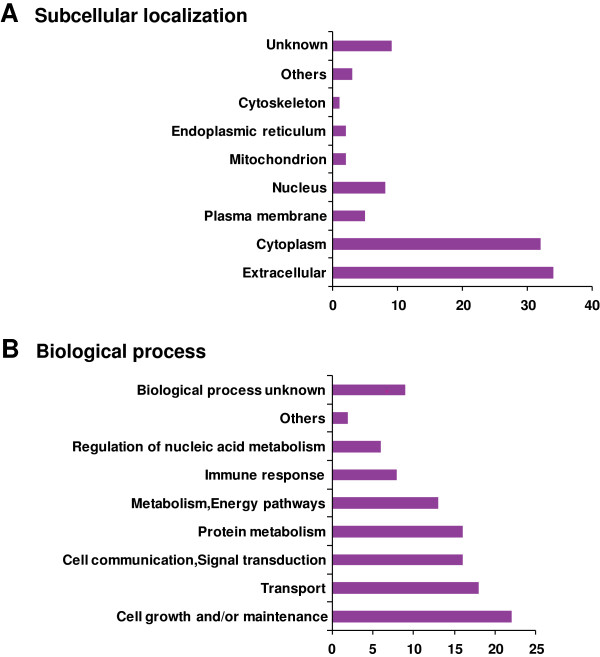
**Gene Ontology**-**based classification of differentially expressed proteins identified from RA and OA synovial fluid. (A)** Subcellular localization **(B)** Biological processes.

Subcellular localization and biological process based classification for the differentially expressed proteins was performed using HPRD [27,28] (http://www.hprd.org) that contains information on human proteins manually curated from the published literature. Signal peptide and domain analysis was also carried out using the data from HPRD. Classification based on the subcellular localization (Figure. 2A) revealed that 34% of the identified proteins were extracellular. Biological process-based (Figure. 2B) categorization showed that a majority of the proteins were involved in cell growth and/or maintenance (22%) and cell communication or signal transduction (18%). Out of 575 identified proteins, 273 possess a signal peptide, 24 have a transmembrane domain and 73 have both a signal peptide and a transmembrane domain.

### Differentially expressed proteins between RA and OA

Out of the 135 differentially expressed proteins, 92 were found to be upregulated and 43 were downregulated in RA synovial fluid ≥3-fold when compared to OA. Several proteins that were earlier reported to be associated with RA and OA were identified in this study confirming the validity of our approach.

### Proteins previously reported to be associated with RA and OA

S100 family of calcium binding proteins, S100A8, S100A9 and S100A12, have been shown to act as proinflammatory mediators in various autoimmune disorders including arthritis [29]. Baillet *et al*. have shown in a proteomic study that these S100 proteins were present at higher concentration in RA synovial fluid than OA synovial fluid and could serve as markers to discriminate between RA and other inflammatory arthritis [30]. In this study, S100A8, S100A9 and S100A12, were upregulated in the synovial fluid of RA patients by 9.1-fold, 29-fold and 33.4-fold, respectively.

Matrix metalloproteinases (MMPs) are zinc dependent extracellular proteolytic enzymes known to play a major role in tissue remodelling under both physiological and pathological conditions [31]. We identified two MMPs, MMP8 and MMP9, to be more abundant in RA synovial fluid (14.4-fold and 3.4-fold, respectively). The levels of these enzymes have already been shown to be elevated in both synovial fluid and sera of RA patients when compared to OA [32]. We also identified a neutrophil elastase inhibitor, serpin peptidase inhibitor, clade B member 1 (SERPINB1) that was upregulated (4.1-fold) in RA synovial fluid. *Serpinb1* knock-out mice have been used as a model to study the role of neutrophil elastase in cellular homeostasis and inflammation [33]. In a comparative proteomic study between RA and OA, SERPINB1 was detected in RA synovial fluid but not in OA synovial fluid [17].

HtrA serine peptidase 1 (HTRA1) is a serine protease that belongs to the family of high temperature requirement family of proteases [34]. Its expression was found to be higher in the articular cartilage vesicles and tissues of OA patients than normal controls [35,36]. It has been shown to be associated with the progression of the articular cartilage degeneration and in the regulation of osteoblast gene expression through the degradation of extracellular matrix proteins including, fibronectin, decorin and matrix Gla [37,38]. Their levels in the synovial fluid of OA patients were found to be significantly elevated than RA and non-arthritic subjects [39]. In our study, it was upregulated in OA synovial fluid by 3.3-fold. Another serine protease identified in this study is the fibroblast activation protein (FAP). FAP has been shown to be expressed by synovial fibroblasts in both RA and OA patients [40]. Its expression has also been demonstrated in several bone tumors including low-grade myofibroblastic sarcoma and osteosarcomas [41]. In this study, it was found to be upregulated in OA synovial fluid by 5-fold.

Myeloperoxidase (MPO), an oxidoreductase enzyme was upregulated by 15.6-fold in RA in the current study. Studies have showed that MPO is highly associated with oxidative stress in the inflamed RA joints. It is present at higher levels in synovial fluid and plasma of RA patients [42]. Lipocalin 2 (LCN2), also known as neutrophil associated gelatinase lipocalin, is expressed in neutrophils Lipocalin 2 has been shown to activate MMP9 and prevent the degradation of MMP9 as well [43,44]. When compared to OA synovial fluid its concentration has been shown to be significantly higher in RA [17,45]. In our study, it was upregulated by 7.4- fold in RA when compared to OA.

Dysregulation of complement proteins is known to contribute to the pathogenesis of OA [46]. In this study, we identified several complement components including complement 4A (C4A), complement component 4 binding protein, alpha (C4BPA) and complement factor I (CFI) to be present at higher levels in OA synovial fluid than RA by 3.3-fold, 5-fold and 3.3-fold, respectively. Recent proteomic studies have demonstrated an aberrant expression of C4A, C4BPA and CFI in OA synovial fluid than synovial fluid from healthy individuals, suggesting their involvement in joint damage [46,47]. Cartilage intermediate layer protein (CILP) is an extracellular matrix protein predominantly expressed by the human articular cartilage [48]. *CILP* has been shown to be associated with the progression of OA [49]. It acts as an autoantigen and contributes to the development of chronic synovitis in OA [50]. Its presence in the healthy and OA synovial fluid has been already demonstrated in a proteomic study [51]. It has been found to be upregulated by 3.3-fold in OA synovial fluid in our study.

### Proteins not previously reported to be associated with RA and OA

**Figure 3:**
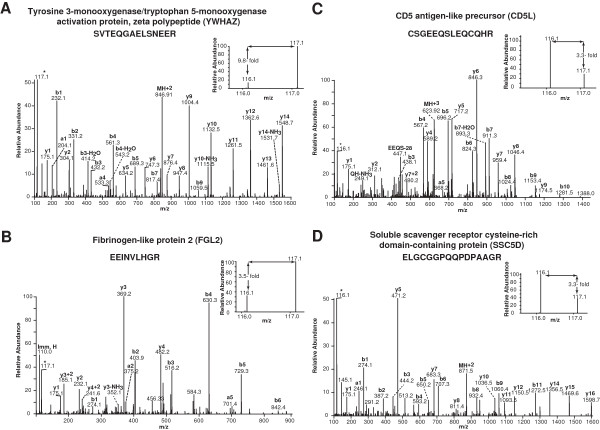
**Representative MS/MS spectra of peptides from novel proteins upregulated in RA and OA synovial fluid.** Novel proteins upregulated in RA: **(A)** Tyrosine 3-monooxygenase/tryptophan 5-monooxygenase activation protein, zeta polypeptide (*YWHAZ*) (9.8-fold), **(B)** Fibrinogen-like protein 2 (*FGL2*) (3.5-fold). Novel proteins upregulated in OA: **(C)** CD5 antigen-like precursor (*CD5L*) (3.3-fold), **(D)** Soluble scavenger receptor cysteine-rich domain-containing protein (*SSC5D*) (3.3-fold).

We identified several differentially expressed proteins which have not been previously associated to the pathogenesis of RA and OA. A partial list of novel proteins upregulated in RA and OA is provided in Table 1 and Table 2 respectively. The representative MS/MS spectra for the novel proteins identified in RA and OA are provided in Figure 3.

**Table 1:** A partial list of novel proteins upregulated in RA

	**Gene symbol**	**Protein**	**Molecular function**	**Cellular component**	**Fold change (RA/OA)**
1	*ARPC1B*	Actin related protein 2/3 complex, subunit 1B, 41 kDa	Cytoskeletal protein binding	Plasma membrane	6.9
2	*CORO1A*	Coronin, actin binding protein, 1A	Cytoskeletal protein binding	Cytoplasm	6.0
3	*YWHAE*	Tyrosine 3-monooxygenase/tryptophan 5-monooxygenase activation protein, epsilon polypeptide	Receptor signaling complex scaffold activity	Cytoplasm	5.2
4	*CTSZ*	Cathepsin Z	Cysteine-type protease activity	Lysosome	3.9
5	*CAPG*	Capping protein (actin filament), gelsolin-like	Cytoskeletal protein binding	Cytoplasm	3.8
6	*FGL2*	Fibrinogen-like 2	Extracellular matrix structural constituent	Extracellular	3.5
7	*TYMP*	Thymidine phosphorylase	Growth factor activity	Extracellular	3.4

**Table 2:** A partial list of novel proteins upregulated in OA

	**Gene symbol**	**Protein**	**Molecular function**	**Cellular component**	**Fold change (OA/RA)**
1	*CRISP3*	Cysteine-rich secretory protein 3	Extracellular matrix structural constituent	Secretory granule	5.0
2	*TTK*	TTK protein kinase	Protein threonine/tyrosine kinase activity	Centrosome	5.0
3	*NOTCH4*	Notch 4	Receptor activity	Plasma membrane	5.0
4	*CD5L*	CD5 molecule-like	Defense/immunity protein activity	Extracellular	3.3
5	*COL15A1*	Collagen, type XV, alpha 1	Extracellular matrix structural constituent	Extracellular	3.3
6	*SSC5D*	Scavenger receptor cysteine rich domain containing (5 domains)	Molecular function unknown	-	3.3
7	*RTN4RL2*	Reticulon 4 receptor-like 2	Receptor activity	Plasma membrane	3.3

Coronin, actin binding protein 1A (CORO1A) is a member of WD repeat protein superfamily expressed in human neutrophils [52]. It has been shown to be distributed at the nascent phagosome and at the leading edge of migrating neutrophils. Functional studies have revealed their role in chemotaxis and phagocytosis in human neutrophils [52]. Studies in mice have demonstrated the importance of *CORO1A* in T cell survival, by stimulating the release of Ca^2+^ from the intracellular reserves upon the activation of T cell receptor [53]. In Th17 CD4 (+) T cells, *CORO1A* served as a positive regulator of TGFβ receptor signaling, thus enhancing the effector functions of these cells [54]. This protein was upregulated by 6-fold in RA when compared to OA in the present study.

Fibroleukin (FGL2) is a serine protease secreted by T lymphocytes [55]. The membrane associated fibroleukin (mFGL2) has prothrombinase activity, whereas the secreted form of fibroleukin has an immunosuppressive effect on T cell proliferation and dendritic cell maturation [56,57]. Studies have shown that *Fgl2* is required for the regulatory activity of T cells and inhibited the development of autoimmune glomerulonephritis [58]. A recent study by Melnyk *et al*. has shown that membrane associated fibroleukin is highly expressed in the arthritic joint space of mice with collagen-induced arthritis (CIA) and its prothrombinase activity contributed to the deposition of fibrin and subsequent inflammation in CIA mice [59]. FGL2 was upregulated in RA patients compared to OA by 3.5-fold.

Different forms of 14-3-3 proteins including 14-3-3 zeta (YWHAZ), 14-3-3 eta (YWHAH), 14-3-3 theta (YWHAQ) and 14-3-3 gamma (YWHAG) were identified in our study. They were upregulated by 9.8-fold, 4.7-fold, 3.2-fold and 3.1-fold, respectively. 14-3-3 proteins are acidic dimeric proteins ubiquitously expressed in eukaryotic cells. These proteins act as phosphoserine/phosphothreonine binding modules. They play significant role in the prevention of apoptosis, initiation and maintenance of DNA damage check points, synchronization of cell adhesion and motility [60].

We identified several proteins upregulated in OA that have not been previously described in the context of OA. CD5 molecule-like protein (CD5L), also known as apoptosis inhibitor expressed by macrophages, is an alternative cell surface ligand for CD5, a glycoprotein expressed on T lymphocytes [61]. It is a soluble protein that belongs to group B scavenger receptor cysteine-rich (SRCR) superfamily [62]. Its expression was detected in the macrophages present in several lymphoid tissues [63]. It was shown to bind to different types of immune cells, suggesting its regulatory role in the immune system [61]. Biochemical studies of this molecule revealed that it is an abundant serum protein and might play a role in the homeostasis of IgM antibodies [62]. *CD5L* has been demonstrated to support the survival of macrophages and enhanced the phagocytic function of macrophages in *Corynebacterium parvum* induced hepatitis [64]. In mice, it was also shown to inhibit the apoptosis of NKT cells and T cells in *C. parvum* induced granulomatous inflammation [65]. This molecule was upregulated in OA by 3.3-fold.

Another newly characterized member of the SRCR superfamily, soluble scavenger receptor cysteine rich domain containing protein (SSC5D) was downregulated by 3.3-fold in RA in our study. Its expression was primarily detected in monocytes/macrophages, T lymphocytes and placenta [66]. Murine Ssc5d has been shown to bind to extracellular matrix constituents such as galectin-1 and laminin as well as to the pathogen-associated molecular patterns in fungi and bacteria, suggesting its function in innate defense and homeostasis of the epithelial surfaces in the host [67].

TTK protein kinase (TTK) is a dual specificity protein kinase essential for centrosome duplication and mitotic progression [68]. Its expression has been shown to be increased by TNF-alpha and IL-2 in human chondrocytes and peripheral blood lymphocytes respectively [69,70]. It was found to be upregulated in OA by 5-fold. In addition, we have also found proteins including, THAP domain-containing protein 4, (THAP4) reticulon 4 receptor-like 2 (RTN4RL2) and leucine rich repeat protein 1 (LRR1) to be upregulated by more than 3-fold in OA.

Although the above-mentioned proteins have been observed to be differentially expressed in the synovial fluid of RA and OA patients, it is possible that they are not really associated with the disease but rather contributed by either individual variation or disease conditions other than arthritis in these patients. This is why, it is necessary to perform validation studies in a larger cohort of samples. In addition, it will be useful to determine if these proteins are detected in synovial or cartilage tissues to understand their association with RA and OA more fully.

### Validation of CAPG by multiple reaction monitoring (MRM) and Western blot

**Figure 4:**
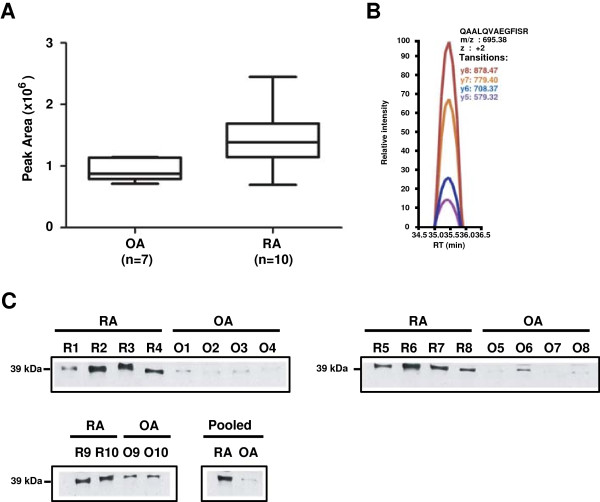
**Validation of CAPG by MRM assay and Western blot. A)** Box and whisker’s plot shows the relative abundance of CAPG in RA compared to OA synovial fluid. **B)** MRM peak traces of the peptide, QAALQVAEGFISR (z = +2, m/z = 695.38) from CAPG (RT: Retention time). **C)** Western blot analysis of CAPG overexpression in RA synovial fluid (n = 10) when compared to OA synovial fluid (n = 10) (Pooled: RA = 10 and OA = 10; R-RA, O-OA).

We adopted a complementary mass spectrometry-based approach, MRM, as well as Western blotting to validate the differential expression of macrophage capping protein, CAPG in RA and OA synovial fluid. CAPG was found to be upregulated in RA synovial fluid by 3.8-fold by iTRAQ quantitation in our discovery study. This protein belongs to the gelsolin family and its interaction with actin is regulated by calcium ions and phosphoinositides [71]. A recent study has speculated its role in mediating a cross-talk between actin cytoskeleton and microtubule-based organelles during cell division [72]. Its association with arthritis has not been reported earlier. CAPG differential expression was confirmed by MRM in RA and OA synovial fluid including the samples that were used for iTRAQ quantitation experiment (Figure 4A and 4B). Western blot analysis also revealed that CAPG was relatively more abundant in individual as well as pooled RA synovial fluid samples (n = 10) when compared to OA synovial fluid samples (n = 10) (Figure 4C). Both MRM and Western blot data were consistent with the LC-MS/MS results.

### Pathway analysis

**Figure 5:**
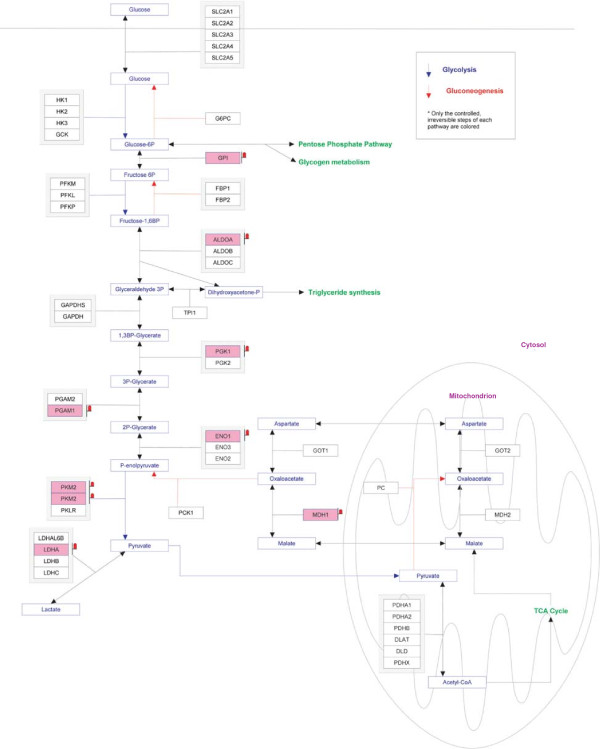
**Glycolytic pathway enriched from GeneSpring analysis.** Proteins upregulated in RA are highlighted in the glycolysis pathway image.

Proteins differentially expressed between RA and OA were subjected to pathway analysis using GeneSpring software suite in order to identify the functional pathways enriched in both the disease conditions. Glycolytic pathway among other pathways showed significant enrichment and we observed that the proteins involved in this pathway to be upregulated in RA when compared to OA. The list of proteins involved in the glycolytic pathway that were upregulated ≥3-fold in RA is provided in Table 3. Figure 5 depicts the glycolytic pathway and highlights the proteins upregulated in RA.

**Table. 3:** List of upregulated proteins in RA involved in glycolytic pathway

	**Gene symbol**	**Protein**	**Fold change (RA/OA)**
1	*HK3*	Hexokinase 3	3.2
2	*GPI*	Glucose-6-phosphate isomerase	4.5
3	*ALDOA*	Aldolase A, fructose-bisphosphate	4.5
4	*PGK1*	Phosphoglycerate kinase 1	4.5
5	*PGAM1*	Phosphoglycerate mutase 1	7.7
6	*ENO1*	Enolase 1, (alpha)	4.7
7	*PKM2*	pyruvate kinase, muscle	3.1
8	*LDHA*	Lactate dehydrogenase A	4.0
9	*MDH1*	Malate dehydrogenase 1, NAD (soluble)	3.2

Glycolysis is a primary glucose metabolic pathway that provides energy for physical activity and coordinates a variety of physiological processes through complex network of signaling pathways [2]. There have been independent reports in the past showing association of different enzymes involved in glycolytic pathway to RA. It has been shown that the activities of two key enzymes of glycolytic pathway, glyceraldehyde 3-phosphate dehydrogenase and lactate dehydrogenase, were higher in RA than the non-rheumatoid synovial tissues [73]. In another study, using magnetic resonance spectroscopy, elevated levels of lactate and lower concentration of glucose were shown to be present in the synovial fluid of RA patients indicating an increased glycolytic activity in their synovium [74]. Hexokinase (HK3) is an enzyme that phosphorylates glucose to form glucose-6 phosphate. Its activity has been reported to be active in the synovial fluid of rheumatoid arthritis patients [75,76]. In our study, HK3 was found to be upregulated by 3.2-fold in RA. Glucose-6-phosphate isomerase (GPI) catalyzes the conversion of glucose-6 phosphate in to fructose-6 phosphate in glycolysis pathway. GPI has been implicated as an autoantigen in RA and their levels were found to be significantly elevated in the serum and synovial fluid of RA patients than non-RA patients [77,78]. Additionally, elevated serum GPI levels have been reported to be more useful in discriminating RA from non-RA patients and thereby in the diagnosis of RA [79]. Autoantibodies to alpha-enolase (ENO1) and aldolase A, fructose-bisphosphate (ALDOA) enzymes involved in the glycolytic pathway were also reported in the sera of RA patients [80,81]. The citrullinated form of ENO1 has been shown to be more prevalent than its native form in the sera of RA patients [82]. In our study, GPI, ENO1 and ALDOA have been found to be upregulated by 4.5-fold, 4.7-fold and 4.5-fold respectively. Our quantitative proteomic analysis is in agreement with previous reports and shows a clear association of glycolytic pathway in RA. In light of this finding, further studies are warranted to investigate this pathway further as a therapeutic option for RA.

### Data availability

The raw data obtained in this study were submitted to public data repositories, Human Proteinpedia (https://www.humanproteinpedia.org) and Tranche (https://www.proteomecommons.org/tranche/). Processed data and the database search results can be downloaded from Human Proteinpedia using the following URL, http://www.humanproteinpedia.org/data_display?exp_id=00702[83]. The following URL can be used to download the raw data from Tranche repository: https://proteomecommons.org/tranche/data-downloader.jsp?fileName=75kcvPwPWE3h7QoO7kH%2FdHfoyAaLSdtAqJQDxLm%2Bosaluq7gID8M4sNfpJ95l9SSJ8ArBv0Om8IBJ%2F0L1kM1tS2BOuIAAAAAAAAKOA%3D%3D.

## Conclusions

In this study, we employed an iTRAQ-based approach to quantitate synovial fluid proteins in RA and OA. A total of 575 proteins were identified and 135 were found to be differentially expressed between RA and OA by ≥3-fold. We identified several proteins that were novel to the study as well as several described earlier in the context of RA and OA. We also confirmed the overexpression of CAPG in RA synovial fluid by MRM as well as by Western blot analysis. Pathway analysis of upregulated proteins in RA revealed a significant enrichment of proteins involved in glycolytic pathway. Further, studies that could unravel the utility of the differentially expressed proteins which in turn might aid in early diagnosis, prognosis as well as in the evaluation of disease progression of RA and OA are warranted.

## Materials and methods

### Sample collection and processing

Approximately 2–4 ml of the synovial fluid samples were aspirated from the affected knees of 10 RA and 10 OA patients in vacutainers (Becton, Dickinson and Company, New Jersey, USA) coated with sodium heparin and stored at 4°C. Only samples collected without any blood contamination during aspiration were included in this study. All the samples were collected from patients who satisfied the American College of Rheumatology criteria for RA and OA [8,84]. The samples were obtained after getting informed consent from the patients and approval from the ethical committees of the Armed Forces Medical College, Pune, India, Fortis Hospitals, Bangalore, India and Command Air Force Hospital, Bangalore, India. Samples were collected from RA and OA patients with an average age of 52 and 65 years, respectively. The samples were centrifuged at 1,500 g at room temperature for 15 minutes and the supernatants were filtered by using 0.22 μm filters (Millipore, Ireland). The filtered samples were stored at -80°C until further analysis.

### Depletion of synovial fluid proteome and iTRAQ labeling

Protein estimation of the synovial fluid samples was carried out using Lowry’s method [85]. Five samples each of OA and RA synovial fluid were pooled separately. The pooled samples were then depleted to remove the 14 most abundant proteins (Albumin, haptoglobin, transferrin, IgA, IgG, alpha 1-antitrypsin, alpha 2-antitrypsin, alpha 1-acid glycoprotein, apolipoprotein A1, apolipoprotein A2, complement C3, IgM, transthyretin and fibrinogen) by using Human 14 multiple affinity removal spin cartridge (Agilent Technologies, Santa Clara, California, USA). The depleted protein was then washed and concentrated using 3 kDa MWCO filters (Amicon, Millipore, Ireland). Approximately 65 μg equivalent of the depleted synovial fluid protein from each group was subjected to trypsin digestion and iTRAQ labeling as described earlier [21]. Briefly, denaturation of the protein was done using 2% SDS followed by reduction and alkylation with reducing agent tris (2-carboxyethyl) phosphine (TCEP) and cysteine blocking agent, methyl methanethiosulfonate (MMTS), respectively. Subsequently the samples were digested with sequencing grade trypsin (Promega, Madison, WI, USA) at 37°C overnight. The tryptic peptides from each group were then labeled with 4-plex iTRAQ reagents (iTRAQ Reagents Multiplex kit, Applied Biosystems, California, USA) as per the manufacturer’s instructions. OA and RA synovial fluid derived tryptic peptides were labeled with 116 and 117 iTRAQ labels respectively. The labeled peptides were then pooled, vacuum-dried and reconstituted in 10 mM KH_2_PO_4_, 20% acetonitrile (pH 2.8) (solvent A) and fractionated by strong cation exchange (SCX) chromatography.

### Strong cation exchange (SCX)-based fractionation

SCX-based fractionation was carried out as described earlier [20]. In brief, the tryptic peptides were fractionated on a PolySULFOETHYL A column (PolyLC, Columbia, MD, USA) with 200 Å, 5 μm, 200 × 2.1 mm dimensions, using an Agilent’s 1200 HPLC-system (Agilent Technologies, Santa Clara, California , USA). A linear gradient of increasing solvent B (350 mM KCl in solvent A, pH 2.8) at a flow rate of 200 μl/min over a period of 70 min was used for fractionation. Peptide fractions were collected using an automated fraction collector. 20 fractions were obtained after fractionation. All the fractions were completely dried and reconstituted in 0.1% trifluoroacetic acid to be further desalted using stage-tips packed with C18 material [86]. Desalted fractions were dried in speedvac and reconstituted in 10 μl of 0.1% TFA prior to LC-MS/MS analysis.

### LC-MS/MS analysis

Tandem mass spectrometric analysis of the iTRAQ labeled peptides were carried out using LTQ-Orbitrap Velos mass spectrometer (Thermo Scientific, Bremen, Germany) interfaced with Easy nanoLC II (previously Proxeon, Thermo Scientific, Bremen, Germany). The LC system consisted of an enrichment column (3 cm × 75 μ) packed with Magic AQ C18 material, 5 μ particle size with 100 Å pore size) and an analytical column (10 cm × 75 μ), Magic AQ C18 material 5 μ particle size, 100 Å pore size) packed using pressure injection cell at 800 psi. Electrospray ionization source was fitted with an emitter tip 8 μm (New Objective, Woburn, MA) and maintained at 2000 V ion spray voltage. Peptide samples were loaded onto an enrichment column in 0.1% formic acid, 5% ACN for 15 min and peptide separation carried out using a linear gradient of 7-35% solvent B (90% ACN in 0.1% formic acid) for 60 minutes at a constant flow rate of 350 nl/min. Data was acquired using Xcalibur 2.1 (Thermo Scientific, Bremen, Germany). The MS spectra were acquired in a data-dependent manner in the m/z range of 350 to 1800 and survey scans were acquired in Orbitrap mass analyzer at a mass resolution of 60,000 at 400 m/z. The MS/MS data was acquired in Orbitrap mass analyzer at a resolution of 15,000 at 400 m/z by targeting top 20 most abundant ions for fragmentation using higher energy collisional dissociation activation at 39% normalised collision energy. Single and unassigned charge state precursor ions were rejected. The dynamic exclusion option was enabled during data acquisition with exclusion duration of 60 seconds. Lock mass option was enabled for real time calibration using polycyclodimethylsiloxane (m/z, 415.12) ions [87].

### Data analysis

Proteome Discoverer Beta Version 1.3 (Thermo Fisher Scientific Inc., Bremen, Germany) was used for database searches. A precursor mass range of 350–8000 Da and a signal to noise of 1.5 were used. A combined Mascot (Mascot version 2.2, Matrix Science) and SEQUEST search was done using the Proteome Discoverer suite (Version 1.3.339, Thermo Scientific, Bremen, Germany) against the NCBI Human RefSeq database 50 containing 33,947 entries with known contaminants. Search parameters included trypsin as the enzyme with maximum 1 missed cleavage allowed; oxidation of methionine was set as a dynamic modification while alkylation at cysteine and iTRAQ modification at N-terminus of the peptide and lysine were set as static modifications. Precursor and fragment mass tolerance were set to 20 ppm and 0.1 Da, respectively. Peptide and protein data were fetched using high peptide confidence and rank one peptide match filters. Reporter ion quantitation node was used for relative expression pattern of proteins based on the relative intensities of reporter ions for the corresponding peptides. The raw data obtained was searched against decoy database to calculate 1% false discovery rate cut-off score [88]. Spectra that matched to the contaminants and those that did not pass the 1% FDR threshold were not considered for analysis.

### Multiple reaction monitoring (MRM)

MRM assays [89] were designed to validate the differentially expressed protein, CAPG in RA and OA synovial fluid. Skyline version 2.1 [90] was used for method development and optimization. The target peptide selected for CAPG was QAALQVAEGFISR (z = +2, m/z = 695.38) and top four transitions monitored included, y8^+^ → 878.47, y7^+^ → 779.40, y6^+^ → 708.37 and y5^+^ → 579.32. The samples were subjected to in-solution digestion as described earlier [91]. All samples were analyzed in triplicate on TSQ Quantum Ultra (Thermo Scientific, San Jose, CA) interfaced with Easy nanoLC II (previously Proxeon, Thermo Scientific, Bremen, Germany). The peptides were enriched on a trap column (5 μm, 75 μm × 2 cm.) with 0.1% formic acid and 5% ACN for 5 minutes and separated on an analytical column (3 μm, 75 μm × 10 cm) with an increasing linear gradient from 5-35% of solvent B (90% ACN in 0.1% formic acid) for 60 min at a constant flow rate of 300 nl/min. Both columns were packed in-house using Magic AQ C18 material (Michrom Bioresources). Spray voltage of 2.5 kV was applied and ion transfer tube was maintained at 275°C. The data was acquired with Q1 and Q3 set at resolution of 0.4 and 0.7 respectively. The collision energy for each transition was optimized with the help of Skyline based on the preliminary results [90].

### Western blot analysis

40 μg of protein from each individual samples of RA (n = 10) and OA (n = 10) synovial fluid was used for Western blot analysis. The primary antibody used was a rabbit anti-human polyclonal antibody for CAPG (Proteintech Group, Inc, Chicago, USA). SDS-PAGE gels were electroblotted on nitrocellulose membrane (Whatman Inc., Maine, USA) at 250 mA for 2 hours using TE-70 ECL semi-dry transfer unit (GE Healthcare, Pittsburgh, USA). The membranes were then blocked with 5% non-fat dry milk and washed with phosphate buffered saline containing 0.05% tween (PBST). The membranes were incubated with primary antibody for 2 hours, washed with PBST and incubated at room temperature for 1 hour in diluted (1:2500) anti-rabbit IgG antibody conjugated with horseradish peroxidase (GE Healthcare, UK).

### Bioinformatics analysis

Gene Ontology (GO)-based [92] analysis was done using Human Protein Reference Database (HPRD) (http://www.hprd.org), which is a GO compliant database [27,28]. Data pertaining to sub-cellular localization, biological processes, domains and motif information associated with the identified proteins were obtained from HPRD.

### GeneSpring Analysis

Pathway Architect module from GeneSpring GX12 was used to identify significantly enriched pathways. Using ‘single experiment analysis’ tool in the pathway architect module the differentially expressed list of genes was searched against publicly available pathways.

## Abbreviations

RA: Rheumatoid arthritis; OA: Osteoarthritis; iTRAQ: isobaric tags for relative and absolute quantitation; SCX: Strong cation exchange; MMPs: Matrix metalloproteinases; CAPG: Macrophage capping protein.

## Competing interests

The authors declare that they have no competing interests.

## Author’s contribution

AP, SS, SM and HG participated in the conception and study design. LB and MB collected the samples and performed the experiments. RSN, SR, MB carried out fractionation and mass spectrometry analysis of the samples. LB, NS, SA and SR were involved in validation experiments. LB prepared the manuscript. LB and YS prepared the manuscript figures. LB, AM, SMS and RR were involved in data analysis and interpretation. VV, MD and NK edited the manuscript. RJ, YLR, TSKP, NS, HG, SM and AP critically read and revised the manuscript. All the authors have read and approved the final manuscript.

## Supplementary Material

Additional file 1: Table S1 List of proteins identified from OA and RA synovial fluid along with the corresponding peptide sequences, coverage, total number of peptides, unique peptides, PSMs, 117/116 (RA/OA) ratio, 117/116 variability, modifications, Xcorr, IonScore, charge, MH+, delta mass (ppm), retention time (RT). Click here for file
